# Masked CKD in hyperthyroidism and reversible CKD status in hypothyroidism

**DOI:** 10.3389/fendo.2022.1048863

**Published:** 2022-11-08

**Authors:** Natsumi Uchiyama-Matsuoka, Kenji Tsuji, Haruhito A. Uchida, Shinji Kitamura, Yoshihiko Itoh, Yuki Nishiyama, Eisaku Morimoto, Satoshi Fujisawa, Tomohiro Terasaka, Takayuki Hara, Kanako Ogura-Ochi, Kenichi Inagaki, Jun Wada

**Affiliations:** ^1^ Department of Nephrology, Rheumatology, Endocrinology and Metabolism, Okayama University Academic Field of Medicine, Dentistry and Pharmaceutical Sciences, Okayama, Japan; ^2^ Department of Chronic Kidney Disease and Cardiovascular Disease, Okayama University Academic field of Medicine, Dentistry and Pharmaceutical Sciences, Okayama, Japan

**Keywords:** chronic kidney disease, masked CKD, reversible CKD, hyperthyroidism, hypothyroidism, eGFR

## Abstract

**Introduction:**

While it is well known that thyroid function may affect kidney function, the transition of the chronic kidney disease (CKD) status before and after treatment for thyroid disorders, as well as the factors affecting this change, remains to be explored. In the present study, we focused on the change in kidney function and their affecting factors during the treatment for both hyperthyroidism and hypothyroidism.

**Methods:**

Eighty-eight patients with hyperthyroidism and fifty-two patients with hypothyroidism were enrolled in a retrospective and longitudinal case series to analyze the changes in kidney function and their affecting factors after treatment for thyroid disorders.

**Results:**

Along with the improvement of thyroid function after treatment, there was a significant decrease in estimated glomerular filtration rate (eGFR) in hyperthyroidism (an average ΔeGFR of -41.1 mL/min/1.73 m^2^) and an increase in eGFR in hypothyroidism (an average ΔeGFR of 7.1 mL/min/1.73 m^2^). The multiple linear regression analysis revealed that sex, eGFR, free thyroxine (FT4) and free triiodothyronine (FT3) could be considered independent explanatory variables for ΔeGFR in hyperthyroidism, while age, eGFR, and FT3 were detected as independent explanatory variables in hypothyroidism. In addition, the stratification by kidney function at two points, pre- and post-treatment for thyroid disorders, revealed that 4.5% of the participants with hyperthyroidism were pre-defined as non-CKD and post-defined as CKD, indicating the presence of “masked” CKD in hyperthyroidism. On the other hand, 13.5% of the participants with hypothyroidism presented pre-defined CKD and post-defined non-CKD, indicating the presence of “reversible” CKD status in hypothyroidism.

**Conclusions:**

We uncovered the population of masked CKD in hyperthyroidism and reversible CKD status in hypothyroidism, thereby re-emphasizing the importance of a follow-up to examine kidney function after treatment for hyperthyroidism and the routine evaluation of thyroid function in CKD patients as well as the appropriate hormone therapy if the patient has hypothyroidism.

## Introduction

It is well known that kidney function and thyroid function are affected by each other (1). Regarding the effects of kidney function on thyroid function, there is an increased prevalence of hypothyroidism and subclinical hypothyroidism in chronic kidney disease (CKD) patients (2, 3), for which several mechanisms were reported, including a decrease in thyrotropin-releasing hormone (TRH) due to uremia (4, 5), thyroid status alterations (i.e., elevation of thyroid-stimulating hormone (TSH) and low thyroxine (T4) and/or triiodothyronine (T3)) by metabolic acidosis (6, 7), and retention of iodine due to a reduction in iodine clearance (8). In addition, there is also an increased prevalence of hypothyroidism in nephrotic syndrome patients due to the urinary loss of thyroid hormones (9). On the other hand, regarding the effect of thyroid disorder on kidney function, there are many reports indicating changes in the kidneys during treatment for thyroid dysfunction in both hyperthyroidism and hypothyroidism. It has been reported that estimated glomerular filtration rate (eGFR) increases after treatment for hypothyroidism (10); conversely, eGFR decreases after the treatment for hyperthyroidism (11). Various mechanisms have been reported, including the effect on renal perfusion pressure through cardiac output and vascular resistance (12, 13), the renin–angiotensin–aldosterone system (RAS), tubular ion transporters (14), the tubulo-glomerular feedback system (15), and creatinine metabolism (16). While these mechanisms include both glomerular filtration rate (GFR)- dependent and GFR-independent pathways, it has been reported that the GFR actually changes before and after treatment for hypothyroidism and hyperthyroidism, even when evaluated with a more accurate GFR by Tc-DTPA (17), indicating the correlation of changes to GFR with thyroid disorder. Thus, the status of CKD might be also altered by treatment for thyroid dysfunction. Using multivariate analysis, in this study, we focused on the possible status transition of CKD before and after treatment for hyperthyroidism and hypothyroidism as well as the clinical factors that may affect the change in eGFR.

## Methods

### Study design and participants

We retrospectively reviewed patients in the division of endocrinology at Okayama University Hospital (Okayama, Japan) between January 2006 and December 2019 *via* electronic-based records. Data collection was completed in 2021. For the analysis of hyperthyroidism, 972 patients were defined as having hyperthyroidism. The exclusion criteria were as follows: 1) age <18 years; 2) defective data for body mass index (BMI); 3) defective data for TSH, free triiodothyronine (FT3), free thyroxine (FT4), serum creatinine (s-Cr), and blood urea nitrogen (BUN) before and after treatment for hyperthyroidism; and 4) longer than 1 year between the pre- and post-treatment examinations. Consequently, 88 patients were enrolled in the hyperthyroid study (**Supplementary Figure S1**). For the analysis of hypothyroidism, 1507 patients were defined as having hypothyroidism, and the exclusion criteria were as follows: 1) age <18 years; 2) defective data for BMI; 3) defective data for TSH, FT3, FT4, s-Cr, and BUN before and after treatment; and 4) longer than 1 year between pre- and post-treatment examinations. Consequently, 52 patients were enrolled in the hypothyroid study (**Supplementary Figure S2**). The latest measurement before treatment was adopted for the pre-examination in both analyses, and the first measurement when FT3 levels reached the normal reference range with TSH levels ≤ 10 µU/mL after treatment was adopted for the post-examination in the hyperthyroidism analysis, as was the first measurement when TSH dropped lower than 4.0 µU/mL after thyroid hormone replacement therapy (THRT). The normal reference range in our institute for TSH was 0.27–4.2 µU/mL, that for FT4 was 0.93–1.7 ng/dL, and that for FT3 was 2.3–4.0 pg/mL. In this study, hyperthyroidism was defined as FT4>1.7 ng/dL and TSH<0.27 µU/L, and hypothyroidism was defined as combined overt hypothyroidism (FT4<0.93 ng/dL and TSH>4.2 µU/L) and subclinical hypothyroidism (0.93≤FT4 ≤ 1.7 ng/dL and TSH>4.2 µU/mL). For the treatment for thyroid disorders, each endocrinologist determined the treatment modalities and doses based on their expertise. The protocol of this study was approved by the ethics committees of Okayama University Hospital Institutional Review Board (accredited ISO9001/2000), Okayama, Japan (approval number: 2101-011), and no written informed consent was obtained because the study was considered exempt. Instead, the contents of the research were posted on our department homepage and the hospital for the public informed consent. In the study, procedures fully adhered to the Declaration of Helsinki.

### Data collection

The following clinical characteristics were collected at the time of treatment for a thyroid disorder: age, sex, BMI, the use of angiotensin-converting-enzyme inhibitor (ACE-i), angiotensin II receptor blocker (ARB), and β-blockers, and hypertension, BUN, and s-Cr and eGFR levels. eGFR was evaluated using the equation developed by the Japanese Society of Nephrology: (eGFR (mL/min/1.73 m^2^) = 194 x s-Cr (mg/dL)^-1.094^ x Age^-0.287^ (x 0.739 for females). CKD was defined as eGFRcre <60 mL/min/1.73 m^2^. Change ratio of eGFR was calculated: Change ratio of eGFR (%) = (post eGFR – pre eGFR)/pre eGFR x 100. ΔeGFR, ΔFT3, and ΔFT4 were calculated: ΔeGFR (mL/min/1.73 m^2^) = post eGFR – pre eGFR, ΔFT3 (pg/mL) = post FT3 – pre FT3, and ΔFT4 (ng/dL) = post FT4 – pre FT4. The presence of hypertension was defined with prior diagnosis or use of anti-hypertensive medications. Thyroid function was measured using an electrochemiluminescence assay (Roche Diagnostics K.K., Cobas 8000).

### Statistical analysis

The statistical analyses were performed by JMP version 14.0.0 (SAS Institute, Inc, Cary, NC) and STATA V15.0 (StataCorp, College Station, TX, USA). All P-values were calculated as two-sided. Significance was defined as p-values less than 0.05. Data were expressed as n (%) for categorical variables and mean ± standard deviation (SD) for continuous variables. Categorical variables were analyzed with a chi-squared test, while continuous variables were compared by using Student’s t-test or Mann–Whitney U test, as appropriate. Correlations among ΔGFR and other variables were evaluated *via* Spearman’s correlation analysis. Multiple linear regression analysis was performed to explore the association of ΔGFR and other variables. A Wilcoxon signed-rank test and paired t test were applied to evaluate the differences in TSH, FT4, FT3, s-Cr, BUN, and eGFR before and after treatment for a thyroid disorder.

## Results

### Study population and clinical characteristics in the hyperthyroidism analysis

Eighty-eight patients met the selection criteria for the analysis (**Supplementary Figure S1**). The characteristics of the participants and laboratory parameters before and after treatment for a thyroid disorder are shown in **Table 1**. The average age was 44 years, and 11% of the patients were men. Along with an improvement in thyroid function after treatment, including an increase in the level of TSH and decreases in the levels of FT4 and FT3, there was a significant decrease in eGFR and an increase in s-Cr levels, indicating decreased kidney function after treatment for hyperthyroidism, similar to previous reports (11, 18, 19). The average of ΔeGFR was -41.1 mL/min/1.73 m^2^ after treatment. On the other hand, there was a significant decrease in the BUN and BUN/Cr ratio after treatment, suggesting a paradoxical laboratory change of BUN during treatment for hyperthyroidism.

**Table 1:** Clinical characteristics before and after treatment in hyperthyroidism.

Clinical parameters	All patients (n = 88)		
Sex (Male), n (%)	10 (11)		
Age (yr)	44 ± 15		
BMI (kg/m^2^)	21.9 ± 4.7		
Hypertension, n (%)	7 (8)		
ACE-i/ARB intake, n (%)	6 (7)		
βblocker intake, n (%)	53 (60)		
Laboratory Results	Before Therapy	After Therapy	P value
TSH (µU/mL)	0.01 ± 0.00	2.72 ± 2.47	p< 0.001^**^
FT4 (ng/dL)	4.80 ± 2.11	1.08 ± 0.73	p< 0.001^**^
FT3 (pg/mL)	15.19 ± 8.21	2.10 ± 0.65	p< 0.001^**^
s-Cr (mg/dL)	0.47 ± 0.12	0.67 ± 0.16	p< 0.001^**^
eGFR (mL/min/1.73m^2^)	123.8 ± 33.6	82.7 ± 17.4	p< 0.001^**^
BUN (mg/dL)	14.3 ± 4.2	13.2 ± 5.0	0.006^**^
BUN/Cr ratio	31.6 ± 10.2	20.0 ± 6.7	p< 0.001^**^

BMI, body mass index; ACE-I, angiotensin–converting enzyme inhibitor; ARB, angiotensin II type 1 receptor blocker; TSH, thyroid-stimulating hormone; FT4, free thyroxine; FT3, free triiodothyronine; s-Cr, serum creatinine; eGFR, estimated glomerular filtration rate; BUN, blood urea nitrogen. P-values were obtained by Wilcoxon signed-rank test or paired t-test. **P < 0.01.

### Analysis of the factors influencing the change of eGFR during treatment for hyperthyroidism

**Figure 1:**
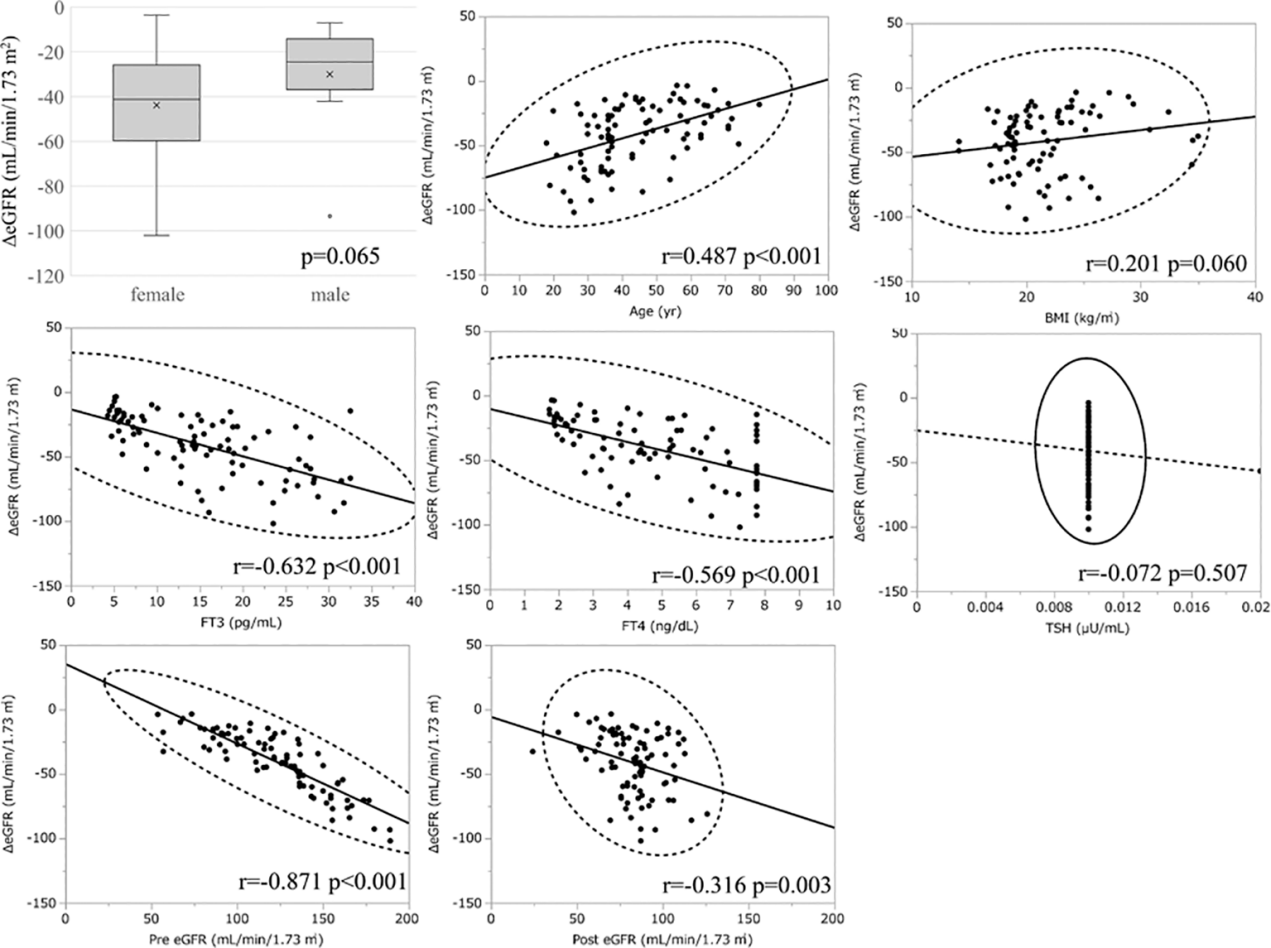
Association between ΔeGFR and variables in hyperthyroidism. ΔeGFR of male and female, and scatter blot analysis between ΔeGFR and variables, age, gender, BMI, post-eGFR, pre-eGFR, FT3, FT4, TSH.

Considering the large decrease in eGFR after treatment for hyperthyroidism, we then explored the variables affecting changes to the eGFR. First, we stratified the participants into groups of ΔeGFR≥-40 (low-decrease group) and ΔeGFR<-40 (high-decrease group), considering that the median ΔeGFR of -37.4 mL/min/1.73 m^2^. The characteristics of the stratified participants are shown in **Table 2**. Patients in the high-decrease group were younger; had higher levels of pre-eGFR, FT4, and FT3; and presented lower levels of pre-s-Cr and lower BMI values compared to patients in the low-decrease group. There were no significant differences in BUN levels between the two groups. We then analyzed the correlation with ΔeGFR. In female patients, the decrease in eGFR was larger than that in male patients (**Figure 1**). We next conducted scatter plot analysis to explore the association between ΔeGFR and variables, which revealed a positive correlation with age and a negative correlation with FT3, FT4, and pre-eGFR but no significant correlation with BMI (**Figure 1**). To further analyze the explanatory variables for ΔeGFR, multiple linear regression analysis was applied (**Table 3**), which detected FT3, FT4, sex, and pre-eGFR as independent explanatory variables for ΔeGFR, while age was not detected after adjustment.

**Table 2:** Characteristics of study participants divided by ΔeGFR (<-40 and ≥-40) in hyperthyroidism.

Characteristics	All patients (n = 88)	ΔeGFR ≥ -40 (n = 46)	ΔeGFR < -40 (n = 42)	P value
Men, %	10 (11)	8 (17)	2 (5)	0.062
Age, yr	44 ± 15	50 ± 14	38 ± 13	<0.001^**^
BMI, kg/m^2^	21.9 ± 4.7	22.9 ± 5.0	20.8 ± 4.1	0.031^**^
Pre sCr, mg/dl	0.47 ± 0.12	0.55 ± 0.12	0.39 ± 0.05	<0.001^**^
Post sCr, mg/dl	0.67 ± 0.16	0.71 ± 0.20	0.63± 0.09	0.074
Pre eGFR, mL/min/1.73 m^2^	123.8 ± 33.6	100.7 ± 22.8	149.1 ± 23.8	<0.001^**^
Post eGFR, mL/min/1.73 m^2^	82.7 ± 17.4	78.0 ± 19.7	87.8 ± 12.7	0.013^*^
ΔeGFR, mL/min/1.73 m^2^	-41.1 ± 23.7	-22.7 ± 9.4	-61.3 ± 17.4	<0.001^**^
Change ratio of eGFR, %	-31 ± 12	-23 ± 10	-41 ± 7	<0.001^**^
Pre BUN, mg/dL	14.3 ± 4.12	14.9 ± 4.9	13.7 ± 3.2	0.345
Post BUN, mg/dL	13.2 ± 5.0	14.2 ± 5.8	12.1 ± 3.5	0.157
TSH, µU/mL	0.01 ± 0.00	0.01 ± 0.00	0.01 ± 0.00	0.306
FT4, ng/dL	4.80 ± 2.11	3.84 ± 1.95	5.86 ± 1.75	<0.001^**^
FT3, pg/mL	15.19 ± 8.21	11.04 ± 6.95	19.74 ± 7.04	<0.001^**^
ΔFT4, ng/dL	-3.72 ± 2.22	-2.79 ± 2.08	-4.74 ± 1.93	<0.001^**^
ΔFT3, pg/mL	-13.09 ± 8.32	-8.90 ± 7.08	-17.69 ± 7.10	<0.001^**^
Hypertension, %	7 (8)	6 (13)	1 (2)	0.065
ACE-i/ARB intake, %	6 (7)	5 (11)	1 (2)	0.115
βblocker intake, %	53 (60)	21 (46)	32 (76)	0.004^**^

BMI, body mass index; TSH, thyroid-stimulating hormone; FT4, free thyroxine; FT3, free triiodothyronine; s-Cr, serum creatinine; eGFR, estimated glomerular filtration rate; BUN, blood urea nitrogen; ACE-I, angiotensin–converting enzyme inhibitor; ARB, angiotensin II type 1 receptor blocker. P-values were obtained by Student’s t-test or Mann–Whitney U test or Pearson’s chi-square test. *P < 0.05, **P < 0.01.

**Table 3A:** Multiple regression analysis of ΔeGFR and variables in hyperthyroidism. (A) Adjusted for sex, age, BMI, eGFR and FT3.

Variable	B	95% CI	β	t	*P-value*
Sex (male)	4.81	1.03 to 8.58	0.13	2.53	0.013^*^
Age, yr	-0.17	-0.38 to 0.04	-0.10	-1.57	0.121
BMI, kg/m^2^	-0.21	-0.73 to 0.32	-0.04	-0.78	0.437
eGFR, mL/min/1.73 m^2^	-0.57	-0.69 to 0.47	-0.82	-10.23	< 0.001^**^
FT3, pg/mL	-0.56	-0.93 to -0.19	-0.19	-3.01	0.004^**^

BMI, body mass index; eGFR, estimated glomerular filtration rate; FT3, free triiodothyronine; B, unstandardized regression coefficient; β, standardized coefficient; CI, confidence interval for B. Model R^2^ = 0.80, adjusted R^2^ = 0.79. *P <0.05, **P<0.01.

**Table 3B:** Adjusted for sex, age, BMI, eGFR and FT4.

Variable	B	95% CI	β	t	*P-value*
Sex (male)	4.31	0.55 to 8.07	0.11	2.28	0.025^*^
Age, yr	-0.18	-0.39 to 0.03	-0.11	-1.69	0.095
BMI, kg/m^2^	-0.22	-0.75 to 0.31	-0.04	-0.82	0.417
eGFR, mL/min/1.73 m^2^	-0.61	-0.72 to -0.50	-0.86	-11.24	< 0.001^**^
FT4, ng/dL	-1.84	-3.19 to -0.50	-0.16	-2.72	0.008^**^

BMI, body mass index; eGFR, estimated glomerular filtration rate; FT4, free thyroxine; B, unstandardized regression coefficient; β, standardized coefficient; CI, confidence interval for B. Model R^2^ = 0.80, adjusted R^2^ = 0.78. *P <0.05, **P<0.01.

### Masked CKD in hyperthyroidism

**Figure 2:**
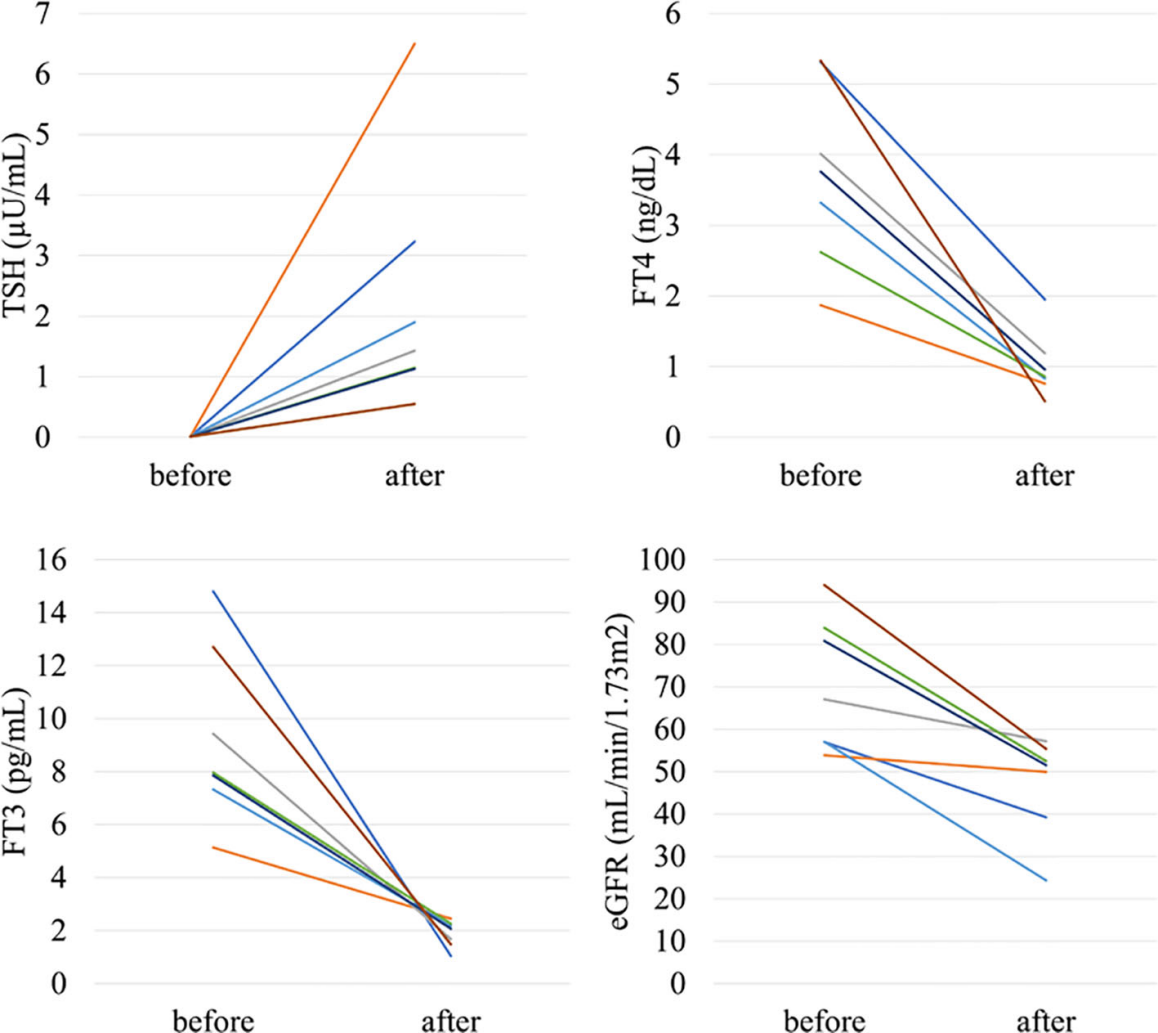
Changes in kidney and thyroid function before and after treatment in “masked” CKD and CKD patients in hyperthyroidism.

**Figure 3:**
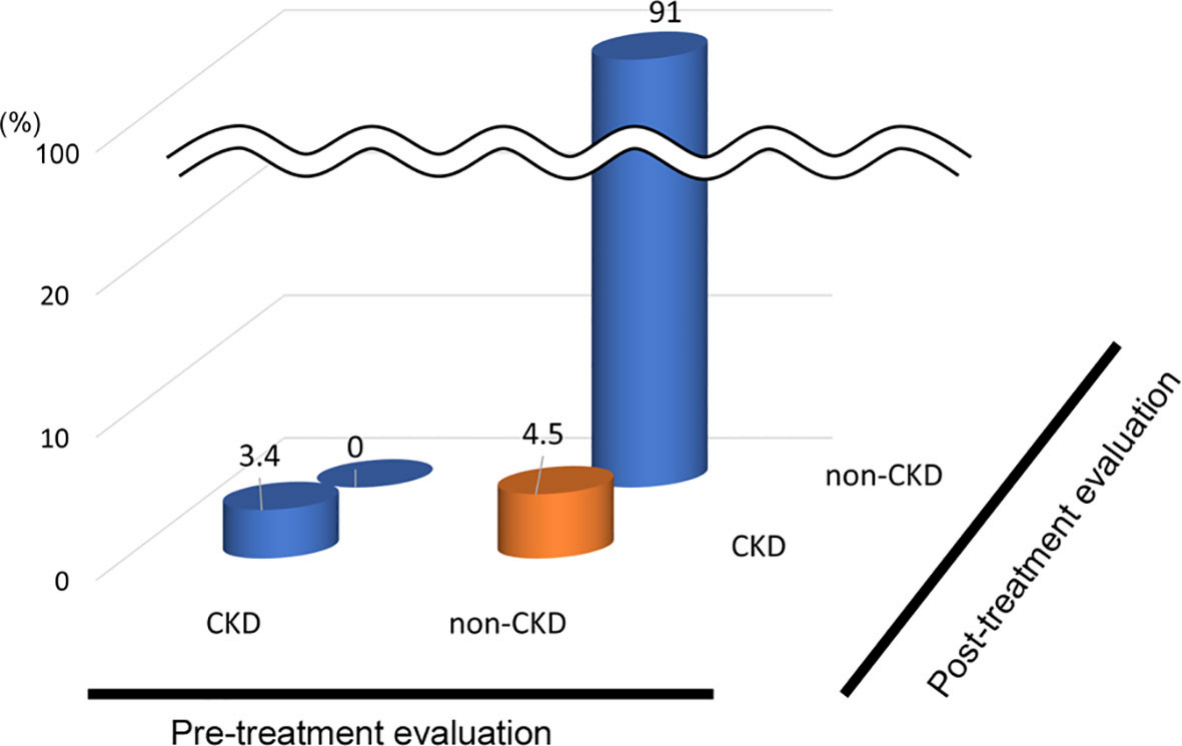
Population of “Masked” CKD in hyperthyroidism. Hyperthyroidism: 4.5% Masked CKD in a total of 7.9% of real-CKD, indicating 57% of CKD was masked).

In the analysis of the eighty-eight patients, we found that four patients who were defined as non-CKD before treatment were defined as CKD after treatment for hyperthyroidism. Laboratory results of kidney and thyroid function before and after treatment revealed that the eGFR values of these patients decreased to less than 60 mL/min/1.73 m^2^ after treatment (**Figure 2**). Since post-eGFR after the treatment for a thyroid disorder may represent the patient’s actual kidney function, these patients were categorized as “masked” CKD under hyperthyroidism. Therefore, we stratified the participants by kidney function at the two points, pre- and post-treatment for hyperthyroidism (**Figure 3**), revealing that 4.5% of participants with hyperthyroidism had masked-CKD. Considering 7.9% of CKD patients were defined *via* post-treatment in hyperthyroidism, 57.1% of the patients with post-defined CKD were masked, suggesting the possibility that CKD may frequently be masked under hyperthyroidism. Surprisingly, three out of four masked CKD patients had pre-eGFR values greater than 80 mL/min/1.73 m^2^. Furthermore, three patients with both pre- and post-defined CKD presented even lower eGFR values after treatment (**Figure 2**), suggesting that particularly careful follow-ups for kidney function are required for patients suffering from hyperthyroidism with CKD. The characteristics of the participants categorized by these groups are shown in **Supplementary Table S1**. Patients in the CKD group were older and had lower levels of pre-eGFR compared to those in the non-CKD group.

### Study population and clinical characteristics in the hypothyroidism analysis

Fifty-two patients met the selection criteria for the analysis (**Supplementary Figure S2**). The characteristics of the participants and the laboratory parameters before and after treatment for a thyroid disorder are shown in **Table 4**. The average age was 58 years and 38% of the patients were men. Along with improvements to thyroid function after treatment, including a decrease in TSH levels and an increase in levels of FT4 and FT3, there was a significant increase in eGFR levels and a decrease in s-Cr levels, indicating decreased kidney function after treatment for hypothyroidism, similar to previous reports (10, 18, 19). The average ΔeGFR was 7.1 mL/min/1.73 m^2^ after treatment. On the other hand, there was no significant difference in BUN after treatment for hypothyroidism, suggesting paradoxical laboratory changes similar to those observed after treatment for hyperthyroidism.

**Table 4:** Clinical characteristics before and after treatment in hypothyroidism.

Clinical parameters	All patients (n=52)		
Sex (male), n (%)	20 (38)		
Age (yr)	58 ± 15		
BMI (kg/m^2^)	22.8 ± 4.0		
Hypertension	13 (25)		
ACE-i/ARB intake n (%)	7 (13)		
Laboratory Results	Before Therapy	After Therapy	*P-value*
TSH (µU/mL)	87.2 ± 175.5	2.2 ± 1.2	0.001^**^
FT4 (ng/dL)	0.65 ± 0.33	1.40 ± 0.36	< 0.001^**^
FT3 (pg/mL)	1.57 ± 0.54	2.65 ± 0.44	< 0.001^**^
s-Cr (mg/dL)	0.95 ± 0.48	0.85 ± 0.48	< 0.001^**^
eGFR (mL/min/1.73m^2^)	64.3 ± 22.5	71.4 ± 22.4	< 0.001^**^
BUN (mg/dL)	17.5 ± 10.6	16.8 ± 11.2	0.298
BUN/Cr ratio	18.7 ± 5.4	20.0 ± 5.9	0.083

BMI, body mass index; ACE-I, angiotensin–converting enzyme inhibitor; ARB, angiotensin II type 1 receptor blocker; TSH, thyroid-stimulating hormone; FT4, free thyroxine; FT3, free triiodothyronine; s-Cr, serum creatinine; eGFR estimated glomerular filtration rate; BUN, blood urea nitrogen. P-values were obtained by Wilcoxon signed-rank test or paired t-test. **P<0.01.

### Analysis of the factors influencing changes in eGFR during treatment for hypothyroidism

**Figure 4:**
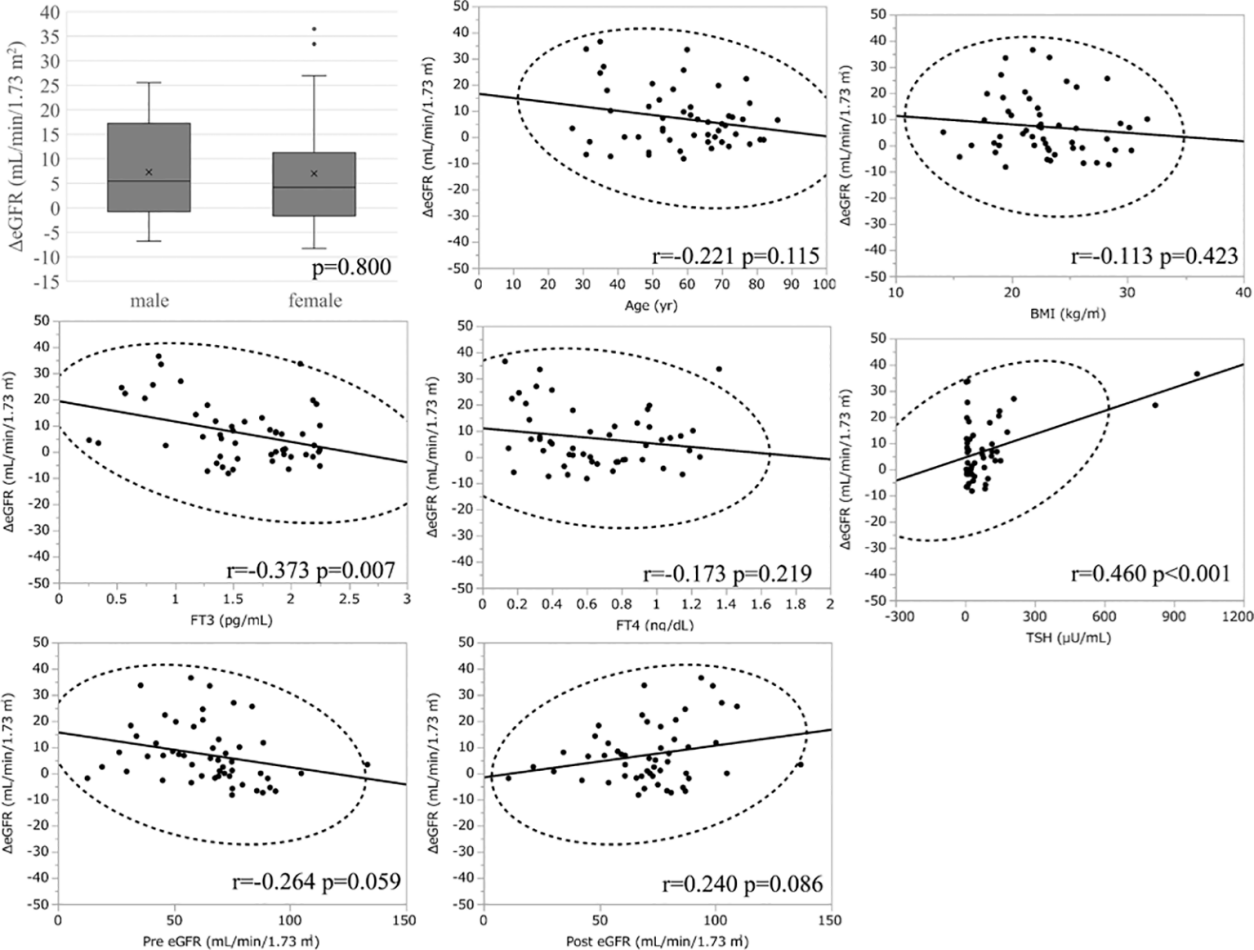
Association between ΔeGFR and variables in hypothyroidism. ΔeGFR of male and female, and scatter blot analysis between ΔeGFR and variables, age, gender, BMI, post-eGFR, pre-eGFR, FT3, FT4, TSH.

Similar to the hyperthyroidism analysis, we next explored variables affecting eGFR changes. First, we stratified the participants into groups of ΔeGFR≥5 (high-elevation group) and ΔeGFR<5 (low-elevation group), considering that the median ΔeGFR of 4.7 mL/min/1.73 m. The characteristics of the stratified participants are shown in **Table 5**. Patients in high-elevation group had higher pre-eGFR, while there was no significant differences regarding age, BMI, post-eGFR, BUN TSH, FT3, or FT4 between the two groups. We then analyzed the correlation with ΔeGFR. There was no significant difference between genders (**Figure 4**). We next conducted a scatter plot analysis to explore the association between ΔeGFR and other variables, which revealed a positive correlation with TSH levels and a negative correlation with FT3 levels (**Figure 4**). To further analyze the explanatory variables for ΔeGFR, multiple linear regression analysis was applied (**Table 6**), which revealed that age and eGFR and FT3 levels were independent explanatory variables for ΔeGFR, while gender and FT4 were not detected as independent explanatory variables, unlike in the analysis for hyperthyroidism. Interestingly, patients with lower pre-eGFR were more likely to show a higher elevation of eGFR, similar to the results for hyperthyroidism.

**Table 5:** Characteristics of study participants divided by ΔeGFR (<5 and ≥5) in hypothyroidism.

Characteristics	All patients (n = 52)	ΔeGFR ≥ 5 (n = 26)	ΔeGFR < 5 (n = 26)	P value
Men, %	20 (38)	10 (38)	10 (38)	1.000
Age, yr	58 ± 15	58 ± 15	58 ± 16	0.891
BMI, kg/m^2^	22.8 ± 4.0	22.6 ± 4.1	23.1 ± 3.9	0.552
Pre sCr, mg/dl	0.95 ± 0.48	0.99 ± 0.36	0.90 ± 0.58	0.026^*^
Post sCr, mg/dl	0.85 ± 0.48	0.78 ± 0.24	0.92 ± 0.64	0.784
Pre eGFR, mL/min/1.73 m^2^	64.3 ± 22.5	57.0 ± 16.6	71.6 ± 25.5	0.006^**^
Post eGFR, mL/min/1.73 m^2^	71.4 ± 22.4	72.8 ± 19.6	70.0 ± 25.2	0.749
ΔeGFR, mL/min/1.73 m^2^	7.1 ± 11.3	15.8 ± 9.5	-1.6 ± 3.6	<0.001^**^
Change ratio of eGFR, %	14 ± 22	30 ± 20	-2 ± 6	<0.001^**^
Pre BUN, mg/dL	17.5 ± 10.6	17.5 ± 8.2	17.5 ± 12.7	0.943
Post BUN, mg/dL	16.8 ± 11.2	15.7 ± 5.5	17.8 ± 14.9	0.641
Pre TSH, µU/mL	87.2 ± 175.5	130.5 ± 239.2	43.8 ± 41.7	0.323
Post TSH, µU/mL	2.2 ± 1.2	1.9 ± 1.3	2.5 ± 1.0	0.067
Pre FT4, ng/dL	0.65 ± 0.33	0.62 ± 0.37	0.68 ± 0.29	0.337
Post FT4, ng/dL	1.40 ± 0.36	1.44 ± 0.40	1.36 ± 0.31	0.126
Pre FT3, pg/mL	1.57 ± 0.54	1.46 ± 0.53	1.69 ± 0.54	0.063
Post FT3, pg/mL	2.65 ± 0.44	2.72 ± 0.55	2.57 ± 0.30	0.191
ΔFT4, ng/dL	0.75 ± 0.48	0.83 ± 0.55	0.68 ± 0.36	0.148
ΔFT3, pg/mL	1.07 ± 0.82	1.26 ± 0.95	0.88 ± 0.62	0.082
Hypertension, %	13 (25)	7 (27)	6 (23)	0.749
ACE-i/ARB intake, %	7 (13)	4 (15)	3 (12)	0.685

BMI, body mass index; TSH, thyroid-stimulating hormone; FT4, free thyroxine; FT3, free triiodothyronine; s-Cr, serum creatinine; eGFR, estimated glomerular filtration rate; BUN, blood urea nitrogen; ACE-I, angiotensin–converting enzyme inhibitor; ARB, angiotensin II type 1 receptor blocker. P-values were obtained by Student’s t-test or Mann–Whitney U test or Pearson’s chi-square test. *P < 0.05, **P < 0.01.

**Table 6A:** Multiple regression analysis of ΔeGFR and variables in hypothyroidism. (A) Adjusted for sex, age, BMI, Post eGFR and FT3.

Variable	B	95% CI	β	t	*P-value*
Sex (male)	0.161	-2.91 to 3.23	0.01	0.11	0.916
Age, yr	-0.219	-0.42 to -0.01	-0.30	-2.14	0.038^*^
BMI, kg/m^2^	-0.304	-1.01 to 0.40	-0.11	-0.87	0.387
eGFR, mL/min/1.73 m^2^	-0.227	-0.36 to -0.10	-0.45	-3.53	0.001^**^
FT3, pg/mL	-8.29	-13.74 to -2.85	-0.40	-3.07	0.004^**^

BMI, body mass index; eGFR, estimated glomerular filtration rate; FT3, free triiodothyronine; B, unstandardized regression coefficient; β, standardized coefficient; CI, confidence interval for B. Model R^2^ = 0.35, adjusted R^2^ = 0.27. *P < 0.05, **P < 0.01.

**Table 6B:** Adjusted for sex, age, BMI, Post eGFR and FT4.

Variable	B	95% CI	β	t	*P-value*
Sex (male)	0.161	-2.44 to 4.11	0.07	0.51	0.613
Age, yr	-0.219	-0.49 to -0.06	-0.37	-2.54	0.015^*^
BMI, kg/m^2^	-0.304	-1.18 to 0.31	-0.15	-1.17	0.248
eGFR, mL/min/1.73 m^2^	-0.227	-0.35 to -0.07	-0.42	-3.07	0.004^**^
FT4, ng/dL	-6.64	-15.97 to 2.68	-0.19	-1.43	0.158

BMI, body mass index; eGFR, estimated glomerular filtration rate; FT4, free thyroxine; B, unstandardized regression coefficient; β, standardized coefficient; CI, confidence interval for B. Model R^2^ = 0.25, adjusted R^2^ = 0.16. *P < 0.05, **P < 0.01.

### Reversible CKD status in hypothyroidism

**Figure 5:**
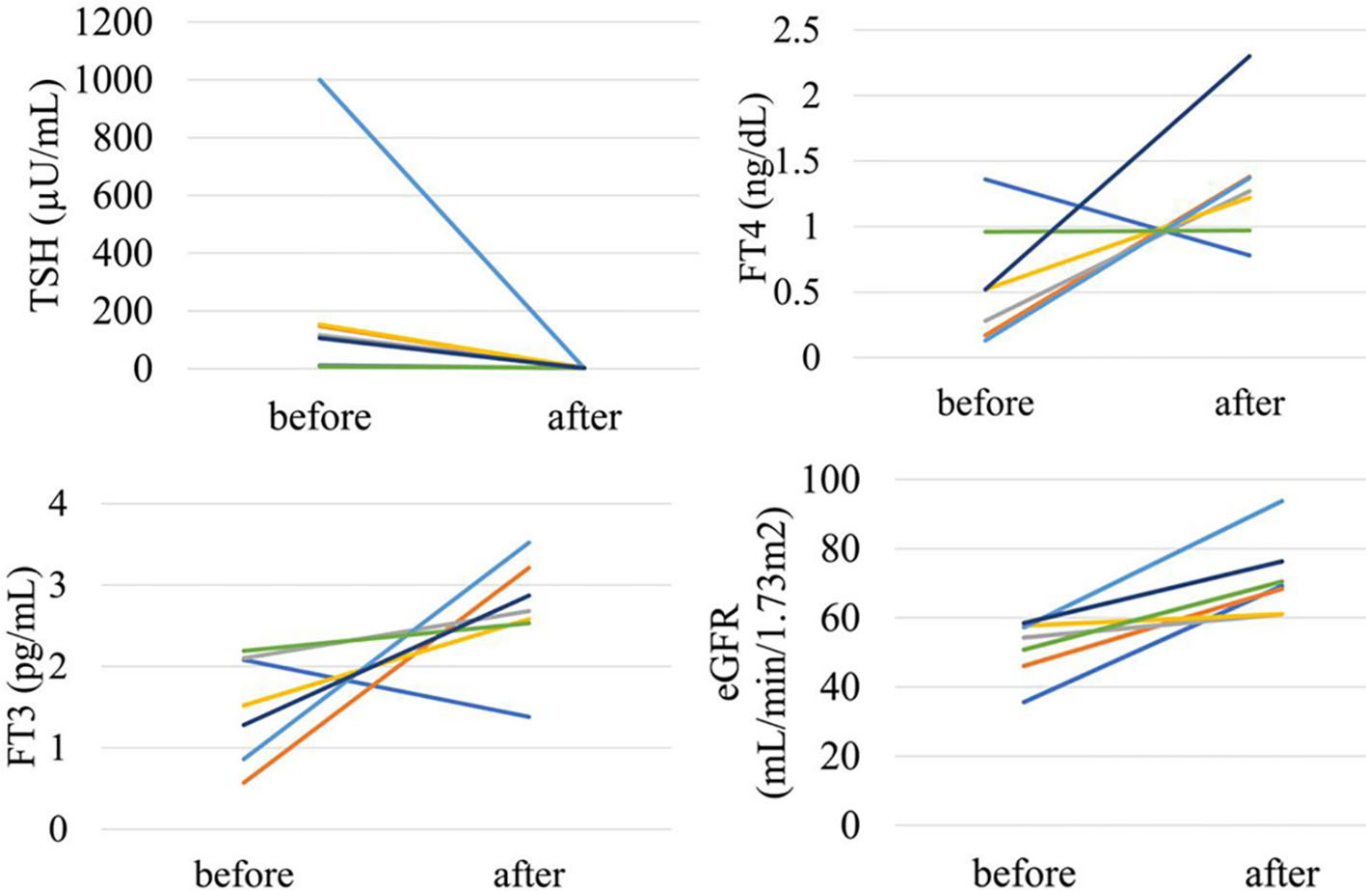
Changes in kidney and thyroid function before and after treatment in “reversible” CKD patients in hypothyroidism.

**Figure 6:**
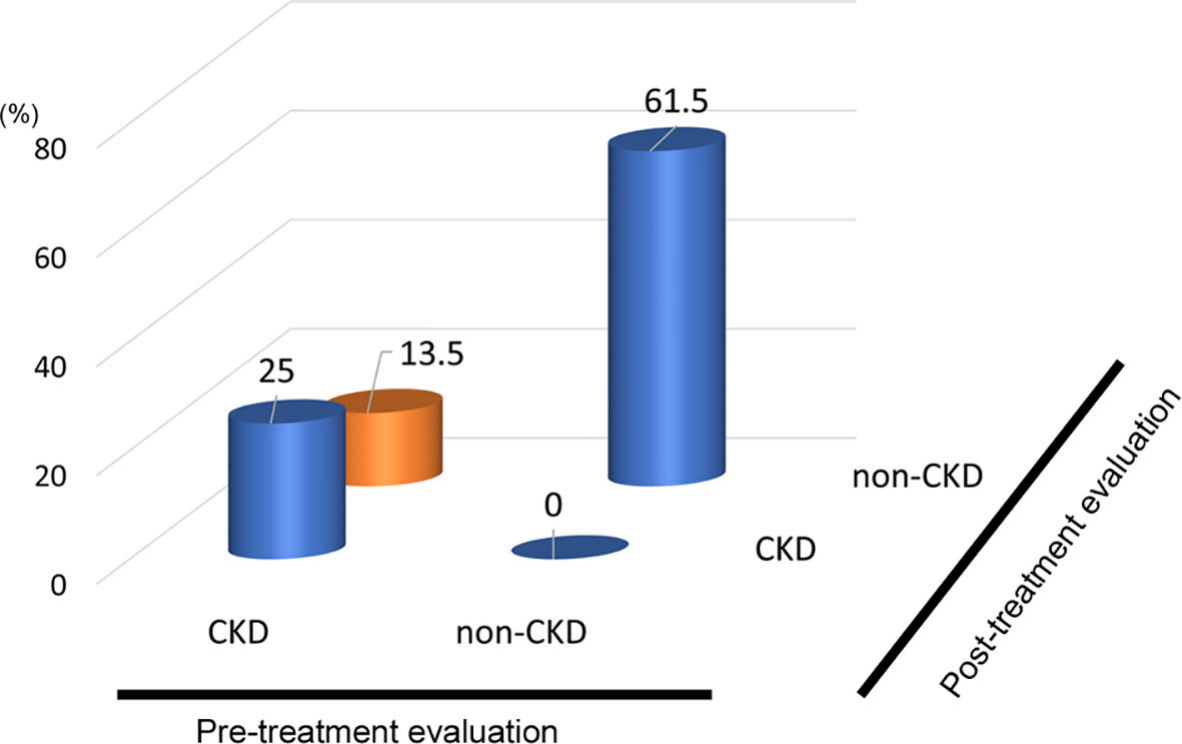
Population of “Reversible” CKD patients in hypothyroidism. Hypothyroidism: 13.5% pseudo-CKD in a total of 38.5% of pre-CKD cases, indicating 35% CKD were reversible.

In the analysis of fifty-two patients, we found seven patients were defined as CKD before treatment and non-CKD after treatment for hypothyroidism. Laboratory results for kidney and thyroid function before and after treatment revealed that the eGFR of these patients increased to more than 60 mL/min/1.73 m^2^ after treatment (**Figure 5**). Again, since post-eGFR after treatment for a thyroid disorder may represent the patient’s actual kidney function, these patients may be categorized as having “reversible” CKD under hypothyroidism. Therefore, we stratified the participants by kidney function at the two points, pre- and post-treatment for hypothyroidism (**Figure 6**), revealing that 13.5% of the participants with hypothyroidism had reversible CKD status. Considering that 38.5% of CKD is defined by pre-treatment in hypothyroidism, 35.0% of the population with pre-defined CKD has a reversible disease, suggesting the possibility that some CKD patients with hypothyroidism may be treatable with THRT. Reversible CKD status was observed among patients whose pre-eGFR values were less than 40 mL/min/1.73 m^2^, defined for CKD stage G3b. In addition, laboratory results of kidney and thyroid functions before and after THRT in the patients with pre- and post-CKD (**Supplementary Figure S3**) showed some elevation in eGFR levels, even though these patients did not recover from CKD, suggesting the importance of THRT for CKD patients with hypothyroidism. The characteristics of these participants stratified by these groups are shown in **Supplementary Table S2**. Patients in the reversible CKD group had lower levels of pre-eGFR and higher levels of ΔeGFR compared to the others.

## Discussion

This study clarified the existence of masked CKD in hyperthyroidism and reversible CKD status in hypothyroidism. Surprisingly, in hyperthyroidism, we showed that out of the 7.9% of cases originally defined as non-CKD, 57% of the cases were defined as CKD after improvement to thyroid dysfunction. Since GFR can be elevated in hyperthyroidism, a follow-up to examine renal function before and after treatment was deemed very important. On the other hand, in hypothyroidism, out of the 38.5% of the cases originally defined as CKD, 35% of the cases were defined as non-CKD after improvements to thyroid dysfunction, indicating a population with reversible CKD status. Especially in the case of CKD with hypothyroidism, renal function can be improved by treating thyroid dysfunction appropriately. Since there are reports that treatment of hypothyroidism slows down the progression of CKD (17) and that hypothyroidism may worsen kidney dysfunction (20), it is necessary to evaluate hypothyroidism in routine analyses of CKD. This is even more important as the prevalence of hypothyroidism increases with the progression of CKD.

Factors affecting the GFR after treatment for thyroid disorders differed between hyperthyroidism and hypothyroidism. In hyperthyroidism, multiple regression analysis showed that eGFR, FT3 and FT4 before treatment significantly affected changes in eGFR, and that the higher the eGFR, FT3 and FT4 before treatment, the greater the decrease in GFR. The effect of age was not considered to be a significant explanatory variable. In hypothyroidism, FT3, but not FT4, was a significant explanatory variable. FT3 is the most metabolically active hormone. T3 and T4 are available in circulation as free hormones, which are biologically active (21). Thyroid hormone molecules can also be activated by conversion from T4 to T3 (22). Various factors related to CKD may affect these processes. It has been reported that FT3 levels are more associated with kidney function compared with FT4 (23), which is in agreement with the result of this study. Although FT4 is more common to be taken in clinical than FT3, FT3 should be checked when considering the relationship with kidney function. In addition, gender was not a significant explanatory variable, and age and pretreatment for eGFR and FT3 had an effect, where the lower the pretreatment for eGFR and FT3, the higher the increase in GFR.

Considering the difference of factors affecting eGFR in hyperthyroidism and hypothyroidism, the mechanisms of eGFR changes induced by thyroid hormone excess or deficiency might be somewhat different. Regarding RAS, hypothyroidism affects the expression, secretion and activity of multiple components of the RAS, which ultimately result in reduced autoregulation of kidney perfusion (24). Conversely, hyperthyroidism increases beta-adrenergic receptors in kidney cortex, and synthesis and secretion of renin by juxtaglomerular cells, which enhance angiotensin-converting enzyme activity, and the activation of RAS and the decrease in the resistance of afferent glomerular arterioles lead to an increase in the glomerular hydrostatic pressure and GFR (25). As for hemodynamics, hypothyroidism reduces cardiac contractility and output which may further impair kidney perfusion (26), while hyperthyroidism increases systolic blood pressure by increasing heart rate, decreasing systemic vascular resistance, raising cardiac output, and increases nitric oxide production, all of which contribute to hyperdynamic circulation, which results in increased renal blood flow (27). In regard to tubular ion transporters, hypothyroidism leads to impaired activity of various tubular ion transporters, which increase sodium and chloride delivery to the distal tubule, thus resulting in increased tubuloglomerular feedback and, pre-glomerular vasoconstriction with a reduction in GFR (1, 14). On the other hand, hyperthyroidism influences the expression and activity of a number of ion channels and transporters by direct binding of thyroid hormone to the promoter region of a transporter gene (28). Collectively, these various factors, some of which are common and some of which are different, may be intricately related to influence changes in eGFR after treatment of thyroid dysfunction.

Interestingly, after treatment for hyperthyroidism, BUN decreased while eGFR decreased. Conversely, after treatment for hypothyroidism, BUN did not decrease significantly while eGFR increased. This result suggests that the thyroid hormone may affect not only the GFR but also BUN independent of the GFR. In fact, an increase in the BUN/Cr ratio was reported in hyperthyroidism (29). In hyperthyroidism, there is both an increase in BUN due to increased urea production caused by retrograde protein catabolism and a decrease in BUN due to an increase in GFR (30). On the other hand, s-Cr levels decrease due to the combination of an increase in GFR, a decrease in muscle mass, and a decrease in Cr caused by an increase in the secretion of Cr from the renal tubules (30). Therefore, it is conceivable that the BUN/Cr ratio may increase under hyperthyroidism. On the other hand, in the examination of hypothyroidism, an increasing tendency of the BUN/Cr ratio was observed before and after treatment, but no significant difference was observed. Because the magnitude of the effect of various mechanisms on BUN and Cr might differ depending on the excess and deficiency of the thyroid hormone, the effect on changes in eGFR can differ between hyperthyroidism and hypothyroidism.

This study has multiple limitations. Firstly, this is a single-center case, and selection bias is unavoidable in cases of hyperthyroidism and hypothyroidism. Secondly, there are many cases excluded, including renal function evaluations, so care should be taken when generalizing the statistical analyses of this study. Thirdly, we did not separately analyze thyroid function diseases such as autoantibodies related to the thyroid gland. It should be considered that the effects on renal function may differ depending on the cause of thyroid disease. Fourthly, we were not able to analyze proteinuria since the data about urinary protein levels were not measured in most cases. Since a change in proteinuria after replacement therapy may help to clarify the mechanism to improve eGFR in reversible CKD status, future study would be desired. Finally, in this study, the evaluation was performed using the eGFR calculation formula suitable for Japanese people. There are multiple eGFR evaluation formulas available, such as CKD-EPI, and it is generally suggested that CKD-EPI is suitable for evaluating the GFR in thyroid disease. It should be noted that the analysis results may differ depending on the evaluation method of renal function. However, changes in GFR before and after treatment of thyroid dysfunction cases are clear, and certain cases were included for masked CKD and reversible CKD cases, even when considering the selection bias. This factor is considered obvious and does not affect our conclusions regarding the concepts of masked CKD and reversible CKD status.

## Conclusion

In this study, we analyzed in detail the changes in eGFR before and after treatment for hyperthyroidism and hypothyroidism, and, with a certain probability, uncovered the presence of masked CKD in hyperthyroidism and reversible CKD status in hypothyroidism cases. We believe that follow-up of analyses on renal function after treatment of hyperthyroidism, evaluations of thyroid function in CKD cases, and appropriate hormone therapy in hypothyroidism cases are important.

## Data availability statement

The raw data supporting the conclusions of this article will be made available by the authors, without undue reservation.

## Ethics statement

The studies involving human participants were reviewed and approved by the ethics committees of Okayama University Hospital Institutional Review Board (accredited ISO9001/2000), Okayama, Japan. Written informed consent for participation was not required for this study in accordance with the national legislation and the institutional requirements.

## Author contributions

U-MN and KT, conception and design of the work, analysis and interpretation of data, and article writing. YI, YN, EM, SF, TT, TH, KO-O, and KI, coordination of the patients’ care and revising the article. HU, SK, and JW interpretation of data, article writing, and final review. All authors contributed to the article and approved the submitted version.

## Funding

The work has been supported by a grant, the Japanese Society for the Promotion of Science (JSPS)/Grant-in-Aid for Young Scientists (20K17283).

## Conflict of interest

The authors declare that the research was conducted in the absence of any commercial or financial relationships that could be construed as a potential conflict of interest.

## Publisher’s note

All claims expressed in this article are solely those of the authors and do not necessarily represent those of their affiliated organizations, or those of the publisher, the editors and the reviewers. Any product that may be evaluated in this article, or claim that may be made by its manufacturer, is not guaranteed or endorsed by the publisher.
