# Supplementary material for: Masked CKD in hyperthyroidism and reversible CKD status in hypothyroidism
Source: Front Endocrinol (Lausanne). 2022 Nov 8;13:1048863. doi: 10.3389/fendo.2022.1048863 (PMC9678909; doi:10.3389/fendo.2022.1048863)
Supplement: Supplementary file 1 [file DataSheet_1.docx]

Supplementary Material

# Supplementary Tables

**Supplementary Table S1. Characteristics of study participants of masked CKD or unmasked CKD and non-CKD in hyperthyroidism.**

| Characteristics | All patients  (n= 88) | Masked and un-masked CKD  (n=7) | Non-CKD  (n=81) | P value |
| --- | --- | --- | --- | --- |
| Men, % | 10 (11) | 0 (0) | 10 (12) | 0.323 |
| Age, yr | 44 ± 15 | 61 ± 8 | 43 ± 14 | 0.002^**^ |
| BMI, kg/m^2^ | 21.9 ± 4.7 | 21.8 ± 3.6 | 21.9 ± 4.8 | 0.823 |
| Pre sCr, mg/dl | 0.47 ± 0.12 | 0.67 ± 0.13 | 0.46 ± 0.11 | <0.001^**^ |
| Post sCr, mg/dl | 0.67 ± 0.16 | 1.00 ± 8.34 | 0.64 ± 14.43 | <0.001^**^ |
| Pre eGFR, mL/min/1.73 m^2^ | 123.8 ± 33.6 | 70.5 ± 15.8 | 128.4 ± 30.6 | <0.001^**^ |
| Post eGFR, mL/min/1.73 m^2^ | 82.7 ± 17.4 | 47.1 ± 11.6 | 85.8 ± 14.1 | <0.001^**^ |
| ΔeGFR, mL/min/1.73 m^2^ | -41.1 ± 23.7 | -23.4 ± 13.0 | -42.7 ± 23.9 | 0.040^*^ |
| Change ratio of eGFR, % | -31 ± 12 | -32 ± 17 | -31 ± 12 | 0.865 |
| Pre BUN, mg/dL | 14.3 ± 4.12 | 19.4 ± 6.9 | 13.9 ± 3.6 | 0.048^*^ |
| Post BUN, mg/dL | 13.2 ± 5.0 | 22.2 ± 9.0 | 12.4 ± 3.6 | 0.003^**^ |
| TSH, µU/mL | 0.01 ± 0.00 | 0.01 ± 0.00 | 0.01 ± 0.00 | 0.801 |
| FT4, ng/dL | 4.80 ± 2.11 | 3.75 ± 1.29 | 4.89 ± 2.15 | 0.199 |
| FT3, pg/mL | 15.19 ± 8.21 | 9.31 ± 3.34 | 15.70 ± 8.32 | 0.061 |
| ΔFT4, ng/dL | -3.72 ± 2.22 | -2.74 ± 1.18 | -3.81 ± 2.28 | 0.287 |
| ΔFT3, pg/mL | -13.09 ± 8.32 | -7.43 ± 3.83 | -13.58 ± 8.43 | 0.076 |
| Hypertension, % | 7 (8) | 1 (14) | 6 (7) | 0.519 |
| ACE-i/ARB intake, % | 6 (7) | 1 (14) | 5 (6) | 0.414 |
| βblocker intake, % | 53 (60) | 3 (43) | 50 (62) | 0.328 |

BMI, body mass index; TSH, thyroid-stimulating hormone; FT4, free thyroxine; FT3, free triiodothyronine; s-Cr, serum creatinine; eGFR, estimated glomerular filtration rate; BUN, blood urea nitrogen; ACE-I, angiotensin–converting enzyme inhibitor; ARB, angiotensin II type 1 receptor blocker. *P*-values were obtained by Student’s t-test or Mann–Whitney U test or Pearson’s chi-square test. *P <0.05, **P<0.01.

**Supplementary Table S2. Characteristics of study participants with or without reversible CKD in hypothyroidism.**

| Characteristics | All patients  (n=52) | reversible CKD  (n=7) | Others  (n=45) | P value |
| --- | --- | --- | --- | --- |
| Men, % | 20 (38) | 2 (29) | 18 (40) | 0.563 |
| Age, yr | 58 ± 15 | 52 ± 18 | 59 ± 15 | 0.355 |
| BMI, kg/m^2^ | 22.8 ± 4.0 | 23.1 ± 3.9 | 22.8 ± 4.0 | 0.936 |
| Pre sCr, mg/dl | 0.95 ± 0.48 | 1.02 ± 0.23 | 0.93 ± 0.51 | 0.051 |
| Post sCr, mg/dl | 0.85 ± 0.48 | 0.75 ± 0.13 | 0.87 ± 0.52 | 0.851 |
| Pre eGFR, mL/min/1.73 m^2^ | 64.3 ± 22.5 | 51.5 ± 8.3 | 66.3 ± 23.4 | 0.032^*^ |
| Post eGFR, mL/min/1.73 m^2^ | 71.4 ± 22.4 | 71.5 ± 11.2 | 71.4 ± 23.7 | 0.748 |
| ΔeGFR, mL/min/1.73 m^2^ | 7.1 ± 11.3 | 20.0 ± 12.4 | 5.1 ± 9.9 | 0.006^**^ |
| Change ratio of eGFR, % | 14 ± 22 | 42 ± 28 | 10 ± 17 | 0.005^**^ |
| Pre BUN, mg/dL | 17.5 ± 10.6 | 19.0 ± 7.60 | 17.3 ± 11.0 | 0.208 |
| Post BUN, mg/dL | 16.8 ± 11.2 | 16.9 ± 6.2 | 16.8 ± 11.8 | 0.494 |
| Pre TSH, µU/mL | 87.2 ± 175.5 | 220.1 ± 348.9 | 66.5 ± 126.0 | 0.068 |
| Post TSH, µU/mL | 2.2 ± 1.2 | 2.6 ± 1.0 | 2.1 ± 1.1 | 0.302 |
| Pre FT4, ng/dL | 0.65 ± 0.33 | 0.56 ± 0.45 | 0.66 ± 0.31 | 0.355 |
| Post FT4, ng/dL | 1.40 ± 0.36 | 1.33 ± 0.45 | 1.41 ± 0.34 | 0.315 |
| Pre FT3, pg/mL | 1.57 ± 0.54 | 1.51 ± 0.64 | 1.58 ± 0.53 | 0.830 |
| Post FT3, pg/mL | 2.65 ± 0.44 | 2.68 ± 0.63 | 2.64 ± 0.40 | 0.369 |
| ΔFT4, ng/dL | 0.75 ± 0.48 | 0.76 ± 0.75 | 0.75 ± 0.41 | 0.555 |
| ΔFT3, pg/mL | 1.07 ± 0.82 | 1.17 ± 1.14 | 1.06 ± 0.74 | 0.727 |
| Hypertension, % | 13 (25) | 2 (29) | 11 (24) | 0.815 |
| ACE-i/ARB intake, % | 7 (13) | 0 (0) | 7 (16) | 0.262 |

BMI, body mass index; TSH, thyroid-stimulating hormone; FT4, free thyroxine; FT3, free triiodothyronine; s-Cr, serum creatinine; eGFR, estimated glomerular filtration rate; BUN, blood urea nitrogen; ACE-I, angiotensin–converting enzyme inhibitor; ARB, angiotensin II type 1 receptor blocker. *P*-values were obtained by Student’s t-test or Mann–Whitney U test or Pearson’s chi-square test. *P <0.05, **P<0.01. *P <0.05, **P<0.01.

# Supplementary Figures


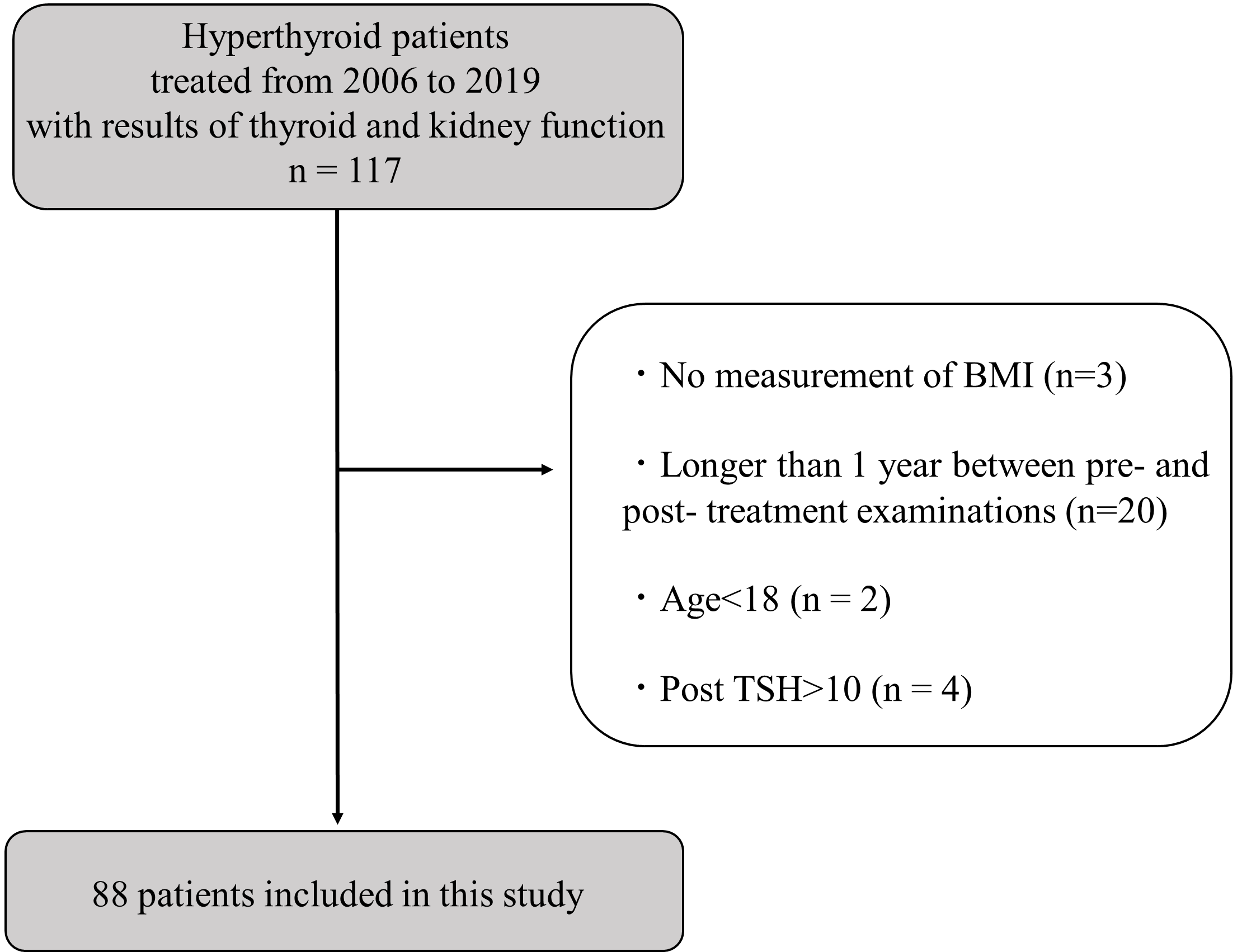


**Supplementary Figure S1.** **Flowchart of study participants for hyperthyroidism.**


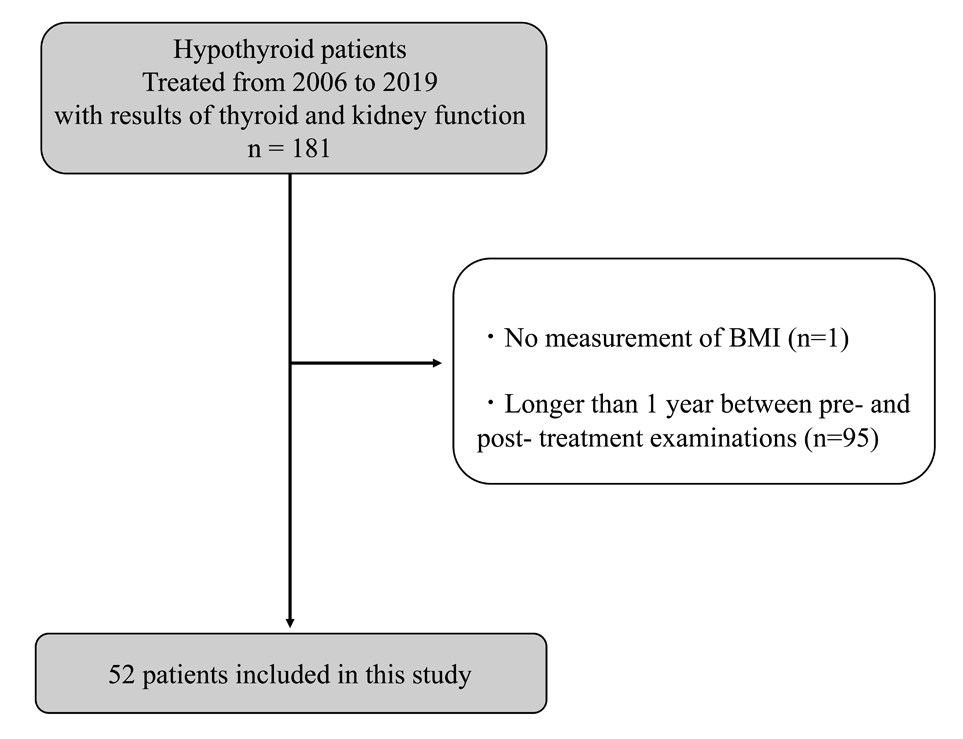


**Supplementary Figure S2.** **Flowchart of study participants for hyperthyroidism.**


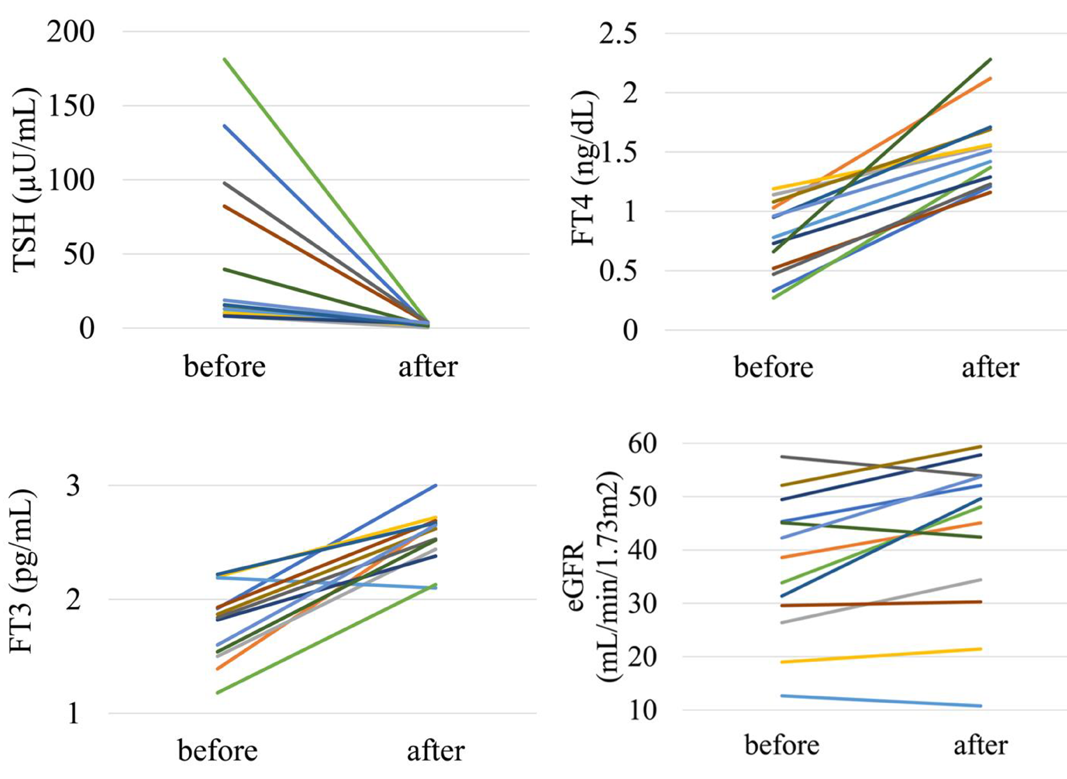


**Supplementary Figure S3.** **Changes in kidney and thyroid function before and after treatment in hypothyroidism with CKD.**

Changes in kidney and thyroid function before and after treatment with CKD patients in hypothyroidism.
